# Intelligent compensation method for measurement errors in optical fiber current sensor caused by temperature variation based on the Levy-Weighted-QPSO-NN algorithm

**DOI:** 10.1371/journal.pone.0363631

**Published:** 2026-07-06

**Authors:** Lin Cheng, Jianyong Luo, Weibin Si, Yanhua Han, Kun Zuo, Haitao Sun, Bo Niu, Shuangzan Ren

**Affiliations:** 1 State Grid Shaanxi Electric Power Co., Ltd. Electric Power Research Institute, Xi’an, China; 2 State Grid Shaanxi Electric Power Co., Ltd, Xi’an, China; 3 State Grid Shaanxi Electric Power Co., Ltd. Ultra-high Voltage Company, Xi’an, China; Southwest Petroleum University, CHINA

## Abstract

Temperature variations significantly degrade the measurement accuracy of fiber optic current sensors (FOCS) in critical power systems applications such as high-voltage transmission and renewable energy integration. To address this, we propose an intelligent error compensation method based on an improved Quantum-behaved Particle Swarm Optimization-Neural Network (Levy-Weighted-QPSO-NN) algorithm. The approach leverages easily measurable state parameters—sensing ring temperature, received optical power, half-wave voltage, SLD temperature, and SLD current—as inputs to predict temperature-induced current ratio difference. Experimental validation involved three sensing rings subjected to temperature cycling (−45 °C to 70 °C), emulating harsh substation environments. The Levy-Weighted-QPSO-NN model achieved 91.11% average prediction accuracy for ratio difference with a correlation coefficient (R²) of 0.9223, outperforming QPSO-NN (85.69%) and Weighted-QPSO-NN (88.31%). Key metrics (MAE: 0.0784; RMSE: 0.0819) confirmed superior stability and accuracy. Robustness testing demonstrated consistent performance across varying population sizes (25–70) and iterations (90–150). Using predicted ratio differences for real-time compensation reduced measurement errors from 0.82% to 0.13%, meeting IEC 61869–6/8 and GB/T standards for Class 0.2S accuracy. This method eliminates reliance on complex hardware modifications, offering a generic, algorithm-driven solution for temperature-dependent FOCS errors.

## 1. Introduction

Fiber optic current sensors, as an innovative alternative to traditional electromagnetic current transformers, are driven by the growing demand for high-precision, high safety, strong anti-interference ability, and wide dynamic range current measurement in power systems [1–3], especially in high voltage, ultra-high voltage transmission, smart grids, new energy grid integration (such as wind power and photovoltaic inverters), and industrial environments with strong electromagnetic interference or flammable and explosive environments.. Its core significance lies in the use of optical fibers and principles (mainly Faraday magneto-optical effect) for non-contact measurement, completely eliminating the problem of magnetic saturation, possessing extremely high electrical insulation performance (intrinsic safety), not affected by electromagnetic interference, having wideband response capability, and a lightweight and easy to integrate structure [4, 5]. Its development has greatly improved the safety, reliability, accuracy, and intelligence level of power system monitoring and control, providing key technical support for smart grid state perception, fault diagnosis, protection control, and digitalization process [6,7]. It is one of the important signs of modernization and intelligent transformation of power equipment.

The research core of improving the measurement accuracy of fiber optic current sensors (FOCT) internationally focuses on tackling key error sources [8]. The main research directions include: temperature stability optimization, suppression of linear birefringence, control of light source and system noise, closed-loop detection and feedback control, advanced signal processing and algorithms, system integration and process optimization [9,10]. For the optimization of temperature stability of fiber optic current sensors, the research focus includes: developing temperature insensitive special magneto-optical materials (such as TGG crystals and specific component glasses) [11], designing innovative optical structures (such as dual path compensation, all fiber Sagnac interferometer structure) [12], using algorithm compensation (feedback correction based on temperature sensors) [13], artificial intelligence modeling prediction compensation [14], exploring temperature self-compensating sensor head packaging technology [15]. This topic has attracted widespread attention from both industry and academic, since the working conditions of temperature variations are extremely common in the application environment of fiber optic current sensor. For example, in ultra-high voltage transmission projects, substations are widely distributed, and their temperature environment may experience extreme conditions of high and low temperatures, resulting in significant temperature changes affecting the measurement of key current parameters such as grounding current by fiber optic current sensors [16,17]. Therefore, it is particularly important to explore the impact of temperature changes on the current measurement ratio and phase difference of fiber optic current sensors. At present, there is an urgent need for a current measurement error compensation method to minimize the impact of temperature changes on measurement accuracy.

Nowadays, the temperature compensation method for fiber optic current sensors (FOCT) mainly focuses on suppressing the Verdet constant temperature drift of magneto-optical materials, reducing the influence of linear birefringence temperature changes, and stabilizing the entire optical system [18]. In terms of material and structural optimization, temperature effects are mainly reduced through the application of special magneto-optical materials, optical structure design, and low birefringence sensing head design and packaging [19]. The relevant hardware compensation technologies include passive optical path compensation and active closed-loop feedback control. However, due to differences in practical application environments, the above methods cannot largely offset the measurement ratio difference caused by temperature changes, and a compensation method with generality and robustness is needed.

In this paper, an innovative method for compensating measurement error of optical fiber current sensor caused by temperature variation is carried out by hybrid intelligent model based on the Levy-Weighted-QPSO-NN algorithm. We chose the state parameters collected by the self-detection module of the fiber optic current sensor as input parameters to predict the ratio difference of the measured current under temperature changes. In view of the advantages of hybrid prediction network structures [20], Levy-Weighted-QPSO-NN algorithm is designed to improve the prediction performance of ratio difference of optical fiber current sensor. Further, the predicted ratio difference is used to provide feedback to the current measurement value and calculate the target current value in reverse, in order to achieve intelligent compensation of the current.

The novelty of this paper lies in the development of an intelligent compensation method for fiber optic current sensor (FOCS) errors using a hybrid Levy-Weighted-QPSO-NN algorithm. This approach uniquely integrates Levy flight search and a weighted average best position calculation into the quantum-behaved particle swarm optimization (QPSO) framework to optimize neural network parameters, significantly enhancing prediction accuracy and convergence efficiency. Unlike traditional hardware-based compensation methods, the proposed technique utilizes easily measurable state parameters as inputs to predict and compensate for temperature-induced ratio differences in real-time, achieving high accuracy (91.11% prediction accuracy, 0.13% post-compensation error) across a wide temperature range (−45 °C to 70 °C). This offers a robust, scalable, and hardware-independent solution that meets stringent industrial standards, marking a significant step forward in intelligent error correction for optical sensing systems.

The reminder of the paper is organized as follows. Section 2 introduces the preliminaries for quantum-behaved particle swarm optimization. Section 3 introduces framework and technical of proposed methodology for intelligent error compensation. Section 4 describes the application of the proposed method. Section 5 presents results and conducts discussions. Finally, conclusions are drawn in Section 6.

## 2. Basic theories for optimization based on quantum-behaved particle swarm

Quantum-behaved Particle Swarm Optimization (QPSO) is an improved swarm intelligence optimization algorithm inspired by the principles of quantum mechanics [21]. It redefines the behavior mechanism of particles by introducing concepts in quantum mechanics (such as quantum state, potential well model and wave function) based on the classical particle swarm optimization (PSO) algorithm. The core idea is to regard particles as being in a quantum state, and their positions are no longer described by a certain trajectory, but by a wave function that satisfies a certain probability density function. Particles move in a potential well (usually a *δ* potential well) formed by the historical optimal position of the group. The probability density of particles appearing at a certain point in space is obtained by solving the Schrödinger equation, and the position of particles is updated using the Monte Carlo random simulation method. The basic update equation of QPSO algorithm can be described by:


$$\begin{array}{c} X_{i,j}\left(t+1\right)=p_{i,j}\left(t\right)\pm \alpha \left|mbest-X_{i,j}\left(t\right)\right|ln\left(\frac{\text{1}}{u_{i,j}\left(t\right)}\right)L_{i,j}\left(t\right) \end{array}$$ (1)

where *mbest* represents the mean best position, *p*_*i*,*j*_(*t*) is the local attractor of particle *i*,*φ*_*j*_(*t*) is, *P*_*ij*_(*t*) is the local best position of particle *i*, *G*_*j*_(*t*) is the global best position.

The QPSO abandons the velocity-position model of the classical PSO by placing particles in a quantum potential well and updating their positions according to the quantum probability model [22], thereby obtaining excellent global search capabilities and the characteristics of avoiding premature convergence. It has a simpler structure, fewer parameters, and theoretically has global convergence. It is particularly suitable for solving challenging and complex optimization problems. It is an important variant in the PSO algorithm family with significantly improved performance.

## 3. The proposed method for intelligent error compensation

### 3.1 Framework of the proposed method

**Fig 1:**
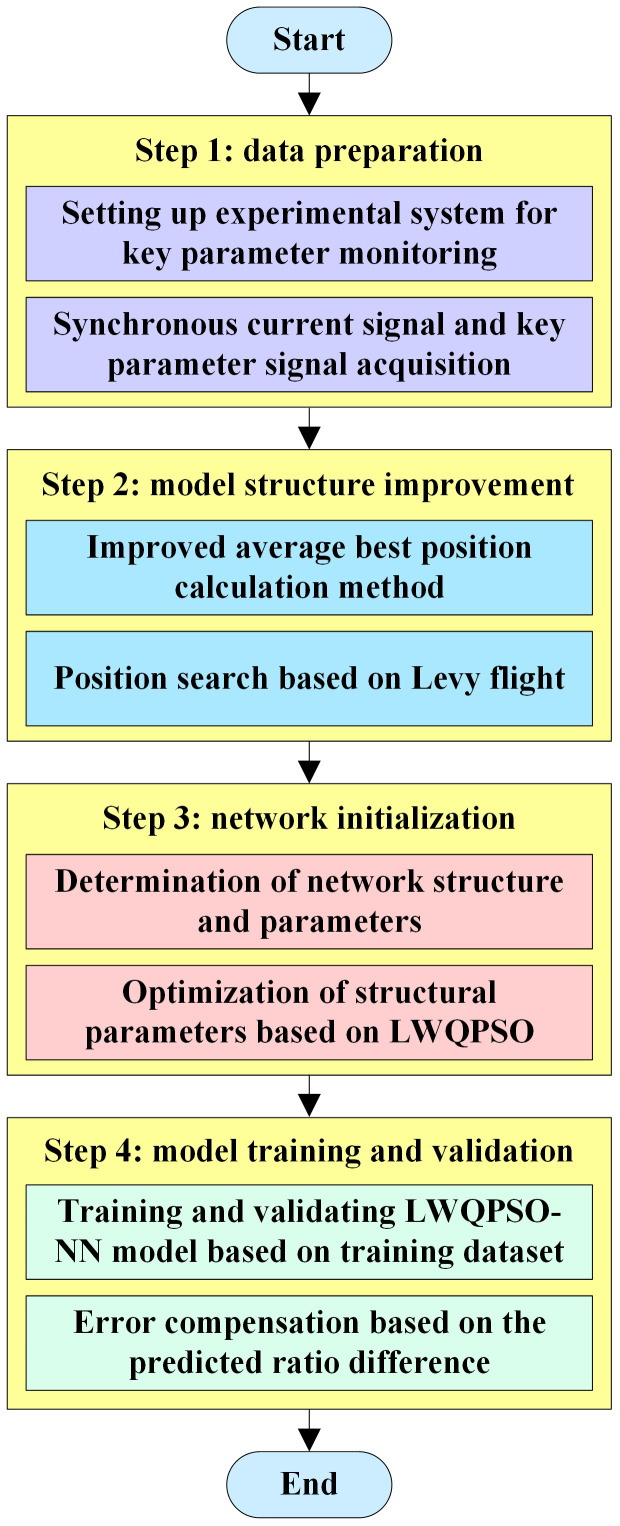
Intelligent error compensation framework based on prediction of measurement ratio difference.

**Fig 2:**
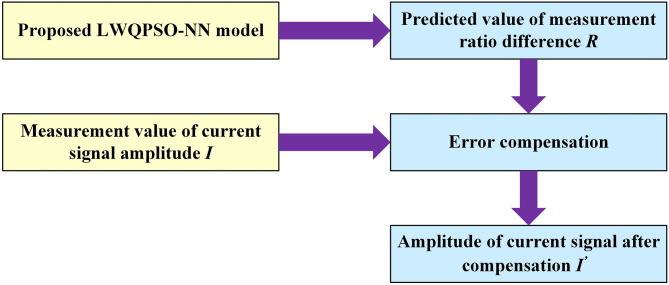
Current amplitude compensation based on predicted measurement ratio difference.


The proposed framework of the proposed method to conduct intelligent error compensation of optical fiber current sensor caused by temperature variation is shown in Fig 1. In the proposed approach, a multi-object algorithm (Levy-Weighted-QPSO) is employed to optimize the structural parameters of neural network for improving the prediction accuracy of measurement ratio difference. The proposed method consists of the following steps:

**Step 1: data preparation**. The experimental system is designed based on close loop system of optical fiber current sensing. In the experimental system, the following parameters: current signal, half-voltage, SLD current, received optical power, sensing ring temperature, and SLD temperature.
**Step 2: model structure improvement.** In this work, two improvement methods are introduced in the traditional system, including Levy flight search and improved average best position calculation. A novel multi-parameter optimization algorithm is proposed name Levy-Weighted-QPSO for obtaining optimal structural parameters of neural network.
**Step 3: network initialization.** Based on the proposed Levy-Weighted-QPSO algorithm, the structural parameters of neural network are obtained for setting up the prediction model of measurement ratio difference. The dataset is constructed based on the synchronous monitoring data.
**Step 4: model training and validation.** According to the constructed Levy-Weighted-QPSO-NN framework, the model is trained and validated to verify whether the expected prediction accuracy and stability can be achieved. Finally, the measured amplitude of current signal is compensated with the predicted ratio difference to output the current value, as shown in Fig 2. The relationship between the measured value of current signal amplitude *I*, predicted measurement ratio different *R*, and amplitude of current signal after compensation *I*^’^ follows the formulation:

$$\begin{array}{c} I^{\prime }=I\cdot \frac{\text{1}}{R+1} \end{array}$$ (2)

### 3.2 Improved QPSO algorithm

This work employs an improved multi-parameter optimization algorithm, namely the improved Levy-Weighted-QPSO algorithm [23]. The improvement of this algorithm is mainly reflected in the following two aspects.

(1) Weighted *mbest* computing method

Faced with the problem of traditional QPSO algorithm easily falling into local optima, the average best position calculation method in traditional QPSO is improved by introducing different individual optimal weight values. The average best position *mbest* is calculated according to Eq. (1) in traditional QPSO, while the improved average best position *mbest*^*’*^ is calculated according to Eq. (1) in IPSO, where *D* represents the number of optimization goals, *N* represents the population size, *pbest* represents the best position of all particles in each dimensions, (*α*_*i*,1_,*α*_*i*,2_,…,*α*_*i*,*D*_) represents the array of weight coefficients with certain regularity. Through this improvement, individual optima closer to the optimal solution will have higher weights, which means they will have a greater impact on the results of the average best position.


$$\begin{array}{c} mbest=\frac{\text{1}}{N}\sum_{i=1}^{N}pbest=\left(\frac{\text{1}}{N}\sum_{i=1}^{N}pbest_{i,1},\frac{\text{1}}{N}\sum_{i=1}^{N}pbest_{i,2},...,\frac{\text{1}}{N}\sum_{i=1}^{N}pbest_{i,D}\right) \end{array}$$ (3)

$$\begin{array}{c} mbest^{\prime }=\frac{\text{1}}{N}\sum_{i=1}^{N}pbest=\left(\frac{\text{1}}{N}\alpha_{i,1}\sum_{i=1}^{N}pbest_{i,1},\frac{\text{1}}{N}\alpha_{i,2}\sum_{i=1}^{N}pbest_{i,2},...,\frac{\text{1}}{N}\alpha_{i,D}\sum_{i=1}^{N}pbest_{i,D}\right) \end{array}$$ (4)

(2) Levy flight for optimal position searching

Levy flight research is an optimization strategy based on the random foraging behavior of organisms in nature, such as the movement patterns of albatrosses and fruit flies [24], which is a random walk model with a step size following a heavy tailed distribution. The mathematical essence of it is that the step sequence satisfies an alpha stable distribution (usually a characteristic exponent), and its probability density function exhibits power-law decay at long distances, alternating between short distance movements and occasional extremely long distance jumps. In the optimization algorithm, Levy flight enhances global exploration capability by generating such random step size updates to the position of the solution [25]. This search strategy has become one of the key technologies for improving the performance of swarm intelligence algorithms, especially suitable for high-dimensional, nonlinear, multimodal function optimization and engineering optimization problems. For the search iteration process, the update equation can be expressed as:


$$\begin{array}{c} \overset{t+1}{\underset{i}{x}}=\overset{t}{\underset{i}{x}}+\alpha ⊕Levy\left(\lambda \right) \end{array}$$ (5)

where *x*_*i*_^*t*^ and *x*_*i*_^*t*+1^ represents flight position of *t*^th^ and *t* + 1^th^ generation respectively; *α* = *α*_0_×(*x*_*i*_^*t*^-*x*_*worst*_), *α*_0_ is constant (0.01), *x*_*worst*_ is *t*^th^ worst particle position. In Eq. (4), the mathematical expression of Levy flight path can be described by:


$$\begin{array}{c} Levy\left(s,\kappa ,\mu \right)=\left\{ \begin{array}{c} @l@\sqrt{\frac{\kappa }{2\pi }}exp\left[-\frac{\kappa }{2\left(s-\mu \right)}\right]\frac{\text{1}}{{\left(s-\mu \right)}^{\frac{\text{3}}{\text{2}}}},0<\mu <s<\infty \\ 0,s\leq 0 \end{array}\right. \end{array}$$ (6)

where *s* represents jump step of Levy flight, *κ* represents the parameter controlling division scale, *μ* represents minimum displacement step.

### 3.3 Input variables of prediction model

In this work, the input variables of the hybrid model are determined as sensing ring temperature, received optical power, half-voltage, SLD temperature, and SLD current. The aforementioned input variables are parameters that are easily synchronously acquired when the fiber optic current sensor performs current measurement in a temperature-varying environment. The dataset is constructed based on the number and types of input variables, and output ratio difference.

### 3.4 Hybrid Levy-Weighted-QPSO-NN model

**Fig 3:**
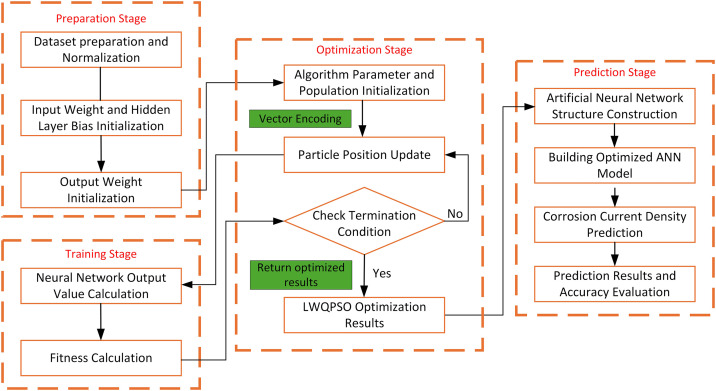
Flow chart of optimization of structural parameters in neural network based on Levy-Weighted-QPSO.

The hybrid Levy-Weighted-QPSO-NN model is constructed based on the optimization of structural parameters through Levy-Weighted-QPSO. The optimized structural parameters include biases term of hidden layer and output layer, and weight from hidden layer to output layer. For the prediction task in this paper, a hidden layer is enough obtain satisfactory prediction accuracy. Flow chart of the optimization process in Levy-Weighted-QPSO is shown in Fig 3.

### 3.5 Model training and testing

The training process of the hybrid Levy-Weighted-QPSO-NN prediction model constructed in this paper is accompanied by continuous updates of structural process, which is carried out by optimization solution based on Levy-Weighted-QPSO algorithm.

### 3.6 Evaluation indexes for prediction performance

In this work, we employ the following six indexes to evaluate the prediction accuracy of measurement ratio difference, including accuracy (AR), mean absolute error (MAE), mean percentage error (MAPE), mean square error (MSE), root mean square error (RMSE), and correlation coefficient *R*^2^. Expressions of these above indexes are shown as follows:


$$\begin{array}{c} AR=\frac{\left|\overset{\prime }{\underset{i}{E}}-E_{i}\right|}{E_{i}}\times 100\% \end{array}$$ (7)

$$\begin{array}{c} MAE=\frac{\sum_{i=1}^{n}\left|\overset{\prime }{\underset{i}{E}}-E_{i}\right|}{n} \end{array}$$ (8)

$$\begin{array}{c} MAPE=\sum_{i=1}^{n}\frac{\left|\overset{\prime }{\underset{i}{E}}-E_{i}\right|}{E_{i}}\cdot \frac{100\%}{n} \end{array}$$ (9)

$$\begin{array}{c} MSE=\frac{\sum_{i=1}^{n}{\left(\overset{\prime }{\underset{i}{E}}-E_{i}\right)}^{\text{2}}}{n} \end{array}$$ (10)

$$\begin{array}{c} RMSE=\sqrt{\frac{\sum_{i=1}^{n}{\left(\overset{\prime }{\underset{i}{E}}-E_{i}\right)}^{\text{2}}}{n}} \end{array}$$ (11)

$$\begin{array}{c} R^{\text{2}}=1-\frac{\sum_{i=1}^{n}{\left(\overset{\prime }{\underset{i}{E}}-E_{i}\right)}^{\text{2}}}{\sum_{i=1}^{n}{\left(E_{i}-\overset{―}{E}\right)}^{\text{2}}} \end{array}$$ (12)

where *E*_*i*_ represents the measured ratio difference, *M*_*i*_^*’*^ represents the predicted ratio difference, $\overset{―}{M}$ represents the average value of ratio difference. Among these indicators, AR is used to directly measure prediction accuracy, MAE and MAPE are used to measure prediction error, *R*^2^ is used to measure similarity between target and predicted ratio difference, and MSE and RMSE are used to measure prediction deviation.

## 4. Application of the measurement error compensation of optical fiber current sensors

### 4.1 Experimental system and data collection

**Fig 4:**
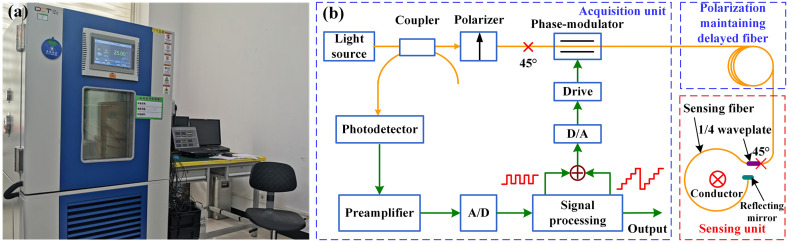
Experimental system of fiber optic current sensor under different environmental temperatures: (a) physical diagram; (b) optical path structure.

Experimental system and optical path of fiber optic current sensor under different ambient temperatures is shown in Fig 4. The sensing ring for fiber optic current is placed inside a temperature chamber to achieve current testing at different ambient temperatures. The fiber optic current transformer is mainly composed of sensing units, acquisition units, and polarization maintaining delay optical cables. The working principle of the fiber optic current transformer is as follows. The light beam emitted by the light source becomes linearly polarized light after passing through the coupler and polarizer. The linearly polarized light reaches the 45° fusion point through the polarization maintaining fiber and is divided into two orthogonal linearly polarized light beams. Then, it undergoes phase modulation through a phase modulator and is transmitted to a 45° fusion point through a polarization maintaining delay line, becoming two pairs of orthogonal linearly polarized light. After passing through a quarter wave plate, it is converted into two beams of circularly polarized light with opposite rotation directions. After entering the sensing fiber, due to the magnetic field generated by the current carrying wire, circularly polarized light undergoes a Faraday phase shift *F* under the action of Faraday effect, resulting in twice the phase difference due to the opposite rotation direction. After passing through the photosensitive fiber ring and reaching the end of the reflective mirror, the original path returns under the reflection of the mirror. At this time, the two modes are exchanged. The returned two beams of light pass through the sensing ring again, and under the action of the magnetic field, the non-reciprocal phase difference due to Faraday effect becomes four times the original. Further, it is transformed into two linearly polarized light beams through a 1/4 wave plate and a 45° fusion point. Finally, the interference signal (light intensity signal carrying current information) enters the photodetector through a coupler and is converted into an electrical signal. The actual value of the current can be demodulated through subsequent preamplifiers, A/D and other signal processing circuits

**Fig 5:**
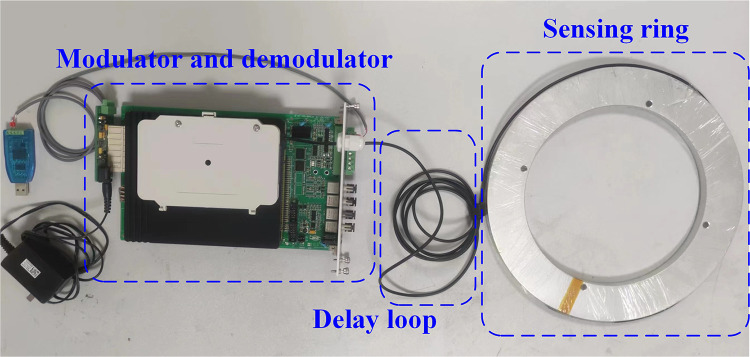
The optical fiber current sensor used for current measurement.

In this experiment, the temperature cycling range is −45°C to 70°C. Three sensing rings with different parameters were used for current measurement experiments at different temperatures. The detailed data results will be analyzed in Section 4.2. The optical fiber current sensor used in the experimental system is shown in Fig 5. The sensing ring was placed in the temperature chamber to obtain the influence of different sensing ring temperatures on the measurement ratio difference, since the sensing ring is mostly likely to be in an environment with strong temperature fluctuations in engineering. The following parameters, including optical module temperature, sensing ring temperature, received optical power, half-wave voltage, SLD temperature, and SLD current, were recorded simultaneously.

### 4.2 Database description and analysis

**Fig 6:**
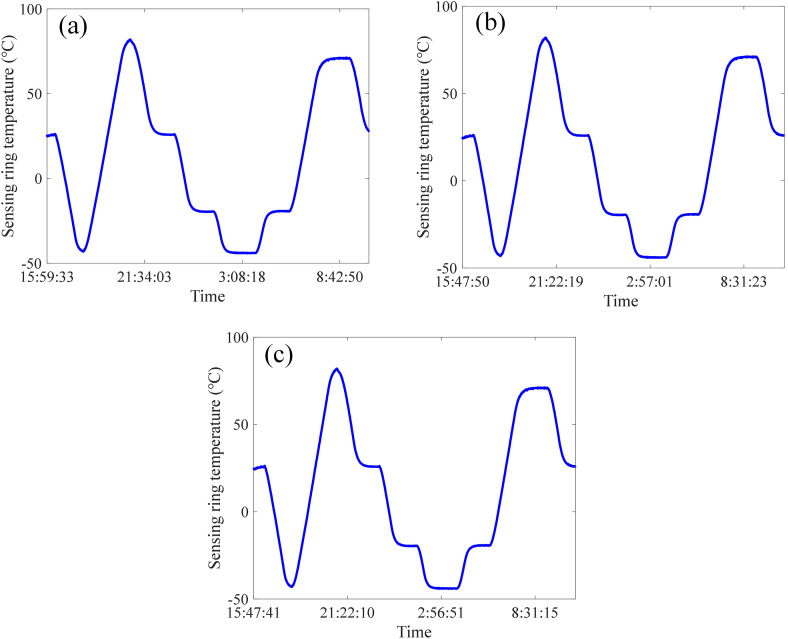
Temperature cycle curves of three sensing optical fiber rings: (a) sensing ring #1; (b) sensing ring #2; (c) sensing ring #3.

Fig 6 shows the temperature cycle curves applied to three sensing optical fiber ring. All three sensing optical fiber rings experienced a cyclic pattern from room temperature to −40°C, then to 70°C and then decreasing repeatedly. The selection of the maximum and minimum temperature for the experiment is based on the temperature range that high-voltage converter stations can experience in different areas.

**Fig 7:**
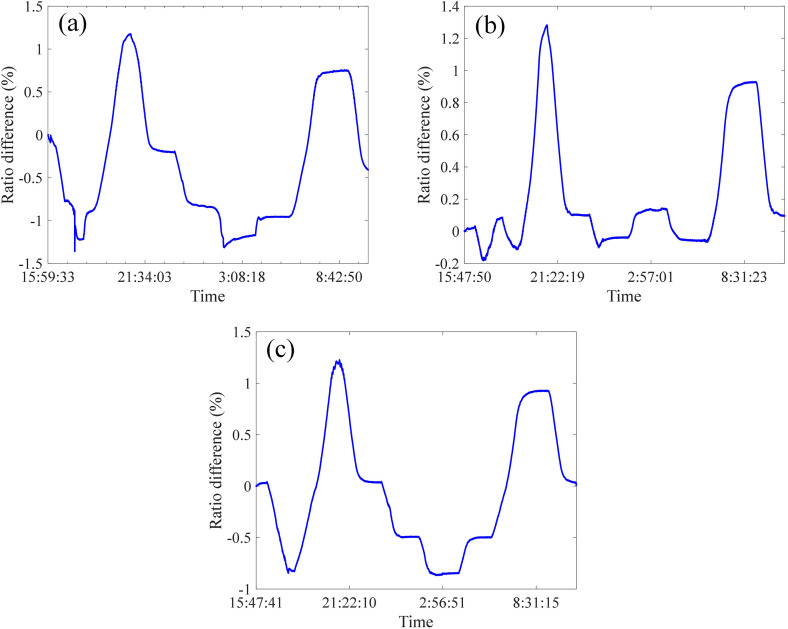
Measurement ratio difference of fiber optical current sensor under temperature cycling: (a) sensing ring #1; (b) sensing ring #2; (c) sensing ring #3.

**Fig 8:**
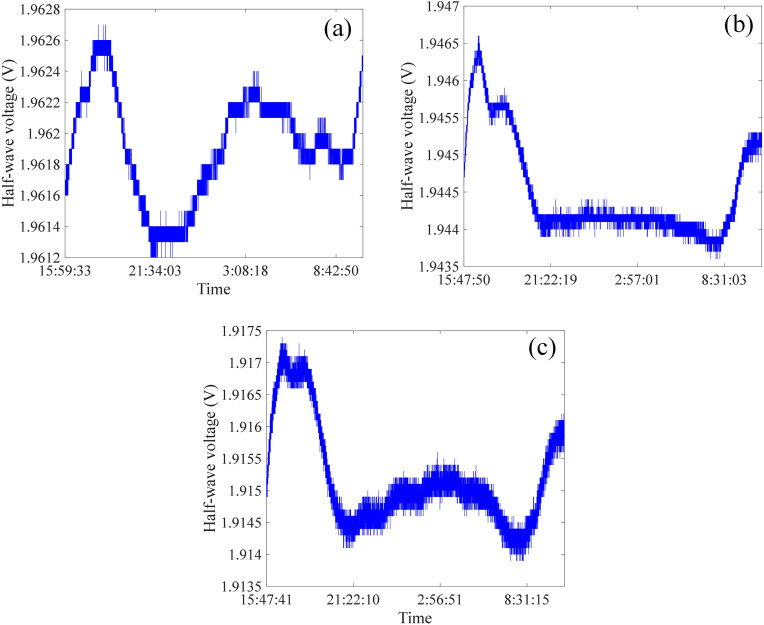
Half-wave voltage of fiber optical current sensor under temperature cycling: (a) sensing ring #1; (b) sensing ring #2; (c) sensing ring #3.

**Fig 9:**
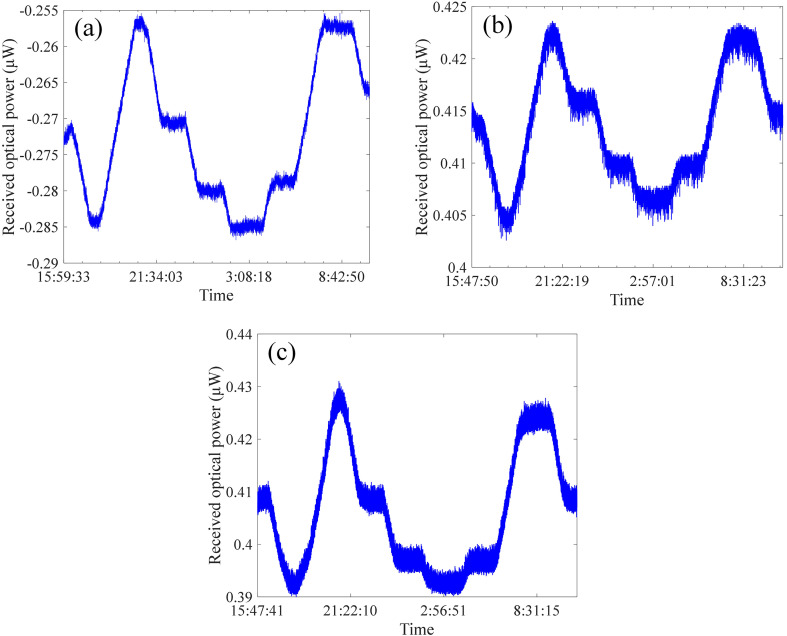
Received optical power of fiber optical current sensor under temperature cycling: (a) sensing ring #1; (b) sensing ring #2; (c) sensing ring #3.

Figs 7–9 show the variation of ratio difference, half-wave voltage and received optical power under temperature cycles. It is seen that there exists strong correlative relationship between ratio difference and temperature of sensing optical fiber ring. Besides, the correlation among ratio difference, received optical power, and half-wave voltage is also observed. Therefore, in this work, the temperature of sensing ring, half-wave voltage, received optical power are all taken as input variables. Besides, the proposed prediction model also takes the SLD temperature and SLD current as input variables.

### 4.3 Model process of ratio difference prediction

#### 4.3.1 Normalization of prediction data.

Normalization is the process of scaling raw data proportionally to a specific range (such as [0,1] or [−1, 1]) or converting it into a standard distribution with a mean of 0 and a standard deviation of 1. Its mathematical essence is to eliminate the differences in dimensions, orders of magnitude, or distribution ranges of different features through linear or nonlinear transformations [26]. Normalization of training and testing data can accelerate model convergence, improve model performance and stability, and enhance algorithm robustness [27]. This work employs linear transformation to process training and test data, as shown in Eq. (13), where where *x*_*i*_*’* represents the *i*^th^ data after normalization, *x*_*i*_ represents the *i*^th^ data before normalization, *x*_max_ represents the maximum value before normalization, *x*_min_ represent the minimum value before normalization.


$$\begin{array}{c} \overset{\prime }{\underset{i}{x}}=\frac{x_{i}-x_{min}}{x_{max}-x_{min}} \end{array}$$ (13)

#### 4.3.2 Fitness function.

During the optimization of structural parameters during Levy-Weighted-QPSO-NN training, fitness function needs to be defined to evaluate the similarity between the target value and the actual value, which is an important component of the multi-parameter optimization algorithm. In this work, the fitness function is defined as given in Eq. (14), where *E*_*i*_ represents measured ratio difference, *E*_*i*_^*’*^ represents the predicted ratio difference.


$$\begin{array}{c} Fitness=\sum_{i=1}^{n}{\left(\overset{\prime }{\underset{i}{E}}-E_{i}\right)}^{\text{2}} \end{array}$$ (14)

#### 4.3.3 Network structure.

**Fig 10:**
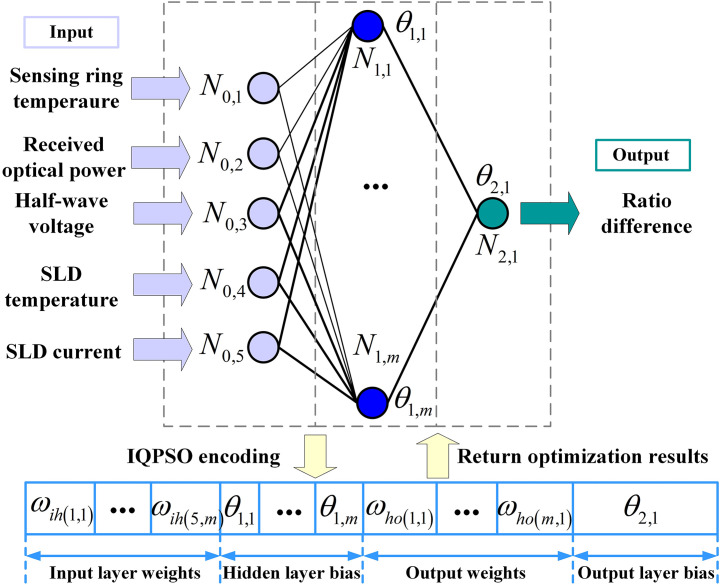
Network structure of hybrid Levy-Weighted-QPSO-NN model for ratio difference prediction.


Network structure of the proposed hybrid prediction model is shown in Fig 10. It needs to mention that the number of neurons in hidden layer is determined according to the Eq. (15), where *m* is the number of neurons in hidden layer, *N*_*s*_ represents the number of samples in training dataset, *N*_*i*_ represents the number of neurons in input layer of hybrid network, *N*_*o*_ represents the number of neurons in output layer of hybrid network. In this network, input variables include sensing ring temperature, received optical power, half-wave voltage, SLD temperature, and SLD current. In addition to this, structural parameters include the connection weights between neurons and the bias term of neurons, which is {*N*_1,1_, *N*_1,2_, ……, *N*_1,*m*_} and {*θ*_1,1_, *θ*_1,2_, ……, *θ*_1,*m*_, *θ*_2,1_} in Fig 10, respectively. The above structural parameters will be formed into a vector as the optimization parameters for Levy-Weighted-QPSO computation. Basic parameters of Levy-Weighted-QPSO-NN algorithm are listed in Table 1.

**Table 1:** Evaluation indicators of ratio difference prediction based on different algorithms.

Algorithm	MAE	MAPE	MSE	RMSE
QPSO-NN	0.1447	16.51%	0.0223	0.1492
Weighted-QPSO-NN	0.1162	13.27%	0.0145	0.1205
Levy-Weighted-QPSO-NN	0.0784	8.89%	0.0067	0.0819

$$\begin{array}{c} m=\frac{N_{s}}{\left[C_{N}⬝\left(N_{i}+N_{o}\right)\right]} \end{array}$$ (15)

## 5. Results and discussion

### 5.1 Discussion of the prediction results

**Fig 11:**
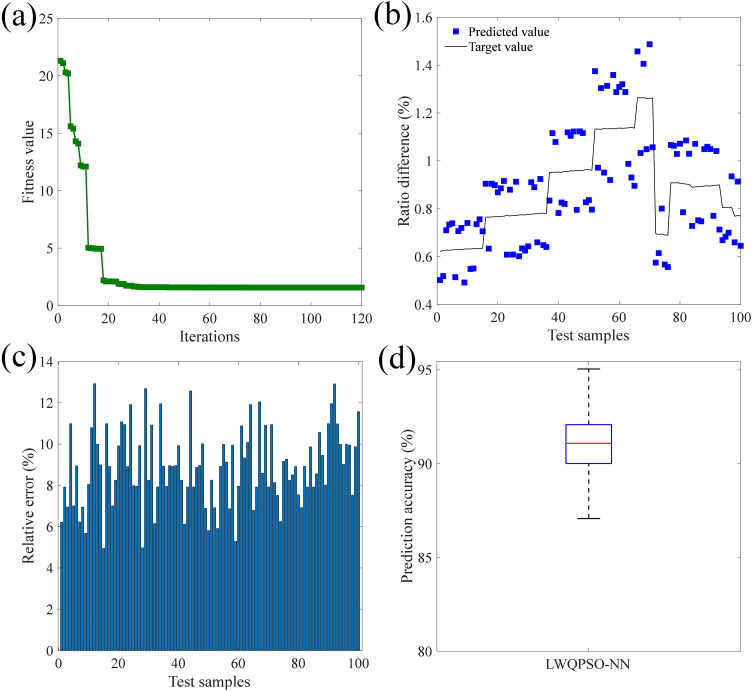
Prediction performance of ratio difference of optical fiber current sensor: (a) optimization convergence curve; (b) prediction results; (c) relative error; (d) distribution of prediction accuracy.

Fig 11 presents the prediction performance of measurement ratio difference of optical fiber current sensor, including the convergence curve of the optimization process, prediction accuracy and absolute error, and the distribution of prediction accuracy. It can be observed from Fig 11(a) that the designed Levy-Weighted-QPSO algorithm can converge quickly and reach a good fitness value when optimizing the structural parameters of the neural network. After more than 20 iterations, the designed Levy-Weighted-QPSO algorithm rapidly converged. After 120 optimization iterations, the optimal fitness value reached 1.5701. It is seen from Fig 11(b) that, based on the proposed Levy-Weighted-QPSO-NN prediction algorithm, the prediction accuracy of ratio difference could reach up to 95.05%, and the average prediction accuracy is 91.11%. The vast majority of the predicted relative errors remain below 8%, as can be seen directly from Fig 11(c). In terms of algorithm accuracy stability, the proposed model’s prediction accuracy has maintained a relatively concentrated distribution of accuracy across multiple tests, with no significant outlier distribution observed. In terms of the algorithm itself, the proposed model can achieve accurate prediction of measurement bias.

### 5.2 Results analysis on prediction performance advantages

**Fig 12:**
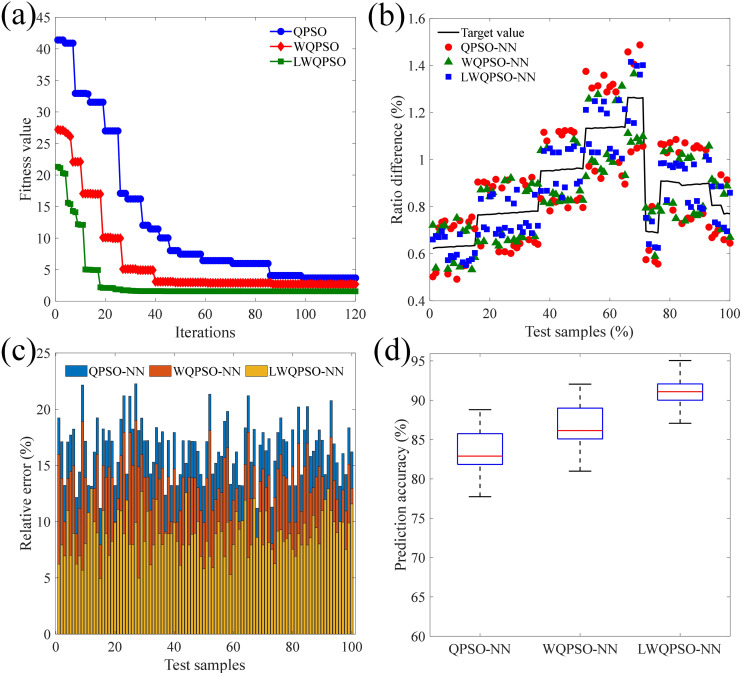
Comparison of prediction performance of ratio difference by different algorithms: (a) optimization convergence curve; (b) prediction results; (c) relative error; (d) distribution of prediction accuracy.

In order to show the effectiveness of the performance improvement method proposed in this work in the traditional QPSO algorithm, as well as the effectiveness in the ratio difference prediction, this section conducts the comparison prediction results of different algorithms, including Levy-Weighted-QPSO-NN, Weighted-QPSO-NN, and QPSO-NN algorithm. These three algorithms are all improved versions based on the quantum behavior particle swarm optimization algorithm to optimize the parameters of neural networks. The core difference lies in the continuous refinement of the particle position update strategy. QPSO-NN mainly uses the quantum mechanics model to replace the velocity-displacement model of the classical PSO, which searches for the global optimal solution through the random expansion and contraction of the “average optimal position” and the “local attraction point”, but its search strategy is relatively homogeneous. WQPSO-NN introduces (usually linearly decreasing) weight factors on the basis of QPSO to dynamically balance the global exploration and local development capabilities, improving the convergence efficiency and accuracy. The analysis in this section will also verify the proposed algorithm improvement measures, achieving a gradual improvement in the performance of the traditional QPSO method. Fig 12 shows the prediction performance comparison among the above algorithms, including optimization convergence, prediction accuracy, and accuracy stability. It is seen from Fig 12(a) that, compared with QPSO and Weighted-QPSO, the proposed Levy-Weighted-QPSO is able to can achieve faster and more accurate optimization convergence. The best fitness value of QPSO, Weighted-QPSO, and Levy-Weighted-QPSO is 3.6800, 2.6876, and 1.5701, respectively. It is observed from Fig 12(b) that, the predicted ratio difference based on Levy-Weighted-QPSO-NN is closer to the actual compared with the results based on QPSO-NN and Weighted-QPSO -NN. Combined with the results shown in Fig 12(c), the average prediction accuracy of QPSO-NN, Weighted-QPSO -NN, and Levy-Weighted-QPSO-NN is 85.69%, 88.31%, and 91.11%, respectively. Besides, it is seen from 12(d) that, the distribution of prediction accuracy of Levy-Weighted-QPSO-NN is more focused compared with QPSO-NN and Weighted-QPSO-NN, indicating that the Levy-Weighted-QPSO-NN model of ratio difference prediction is more stable.

**Fig 13:**
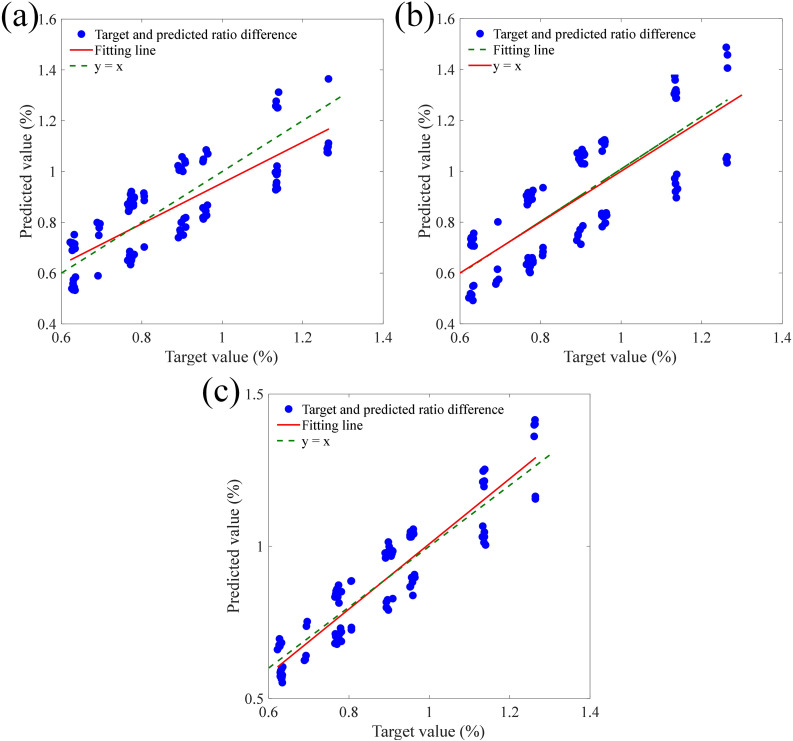
Correlation analysis between predicted ratio difference and target ratio difference based on different algorithms: (a) QPSO-NN; (b) Weighted-QPSO-NN; (c) Levy-Weighted-QPSO-NN.

Fig 13 shows the correlation relationship between actual value and prediction value based on QPSO-NN, Weighted-QPSO-NN, and Levy-Weighted-QPSO-NN model. The correlation coefficient of QPSO-NN, Weighted-QPSO-NN, and Levy-Weighted-QPSO-NN is 0.7824, 0.7903, and 0.9223, respectively. Besides, the MAE, MAPE, MSE, and RMSE between actual value and predicted value is also listed in Table 1. Based on the calculation results of various indicators, the proposed method comprehensively demonstrates its performance advantages in the prediction of measurement ratio difference of fiber optical current sensor.

### 5.3 Robustness analysis on the prediction performance

The analysis of the algorithm’s stability and robustness is directly motivated by practical application concerns. In real-world deployments, the hyperparameters of an intelligent model (like population size and iteration count) may not be perfectly fine-tuned for every individual sensor unit or may need to be adjusted for computational efficiency. By demonstrating that the proposed Levy-Weighted-QPSO-NN model maintains consistent prediction accuracy across a range of these parameters, we prove its inherent reliability and reduced sensitivity to configuration variances. This robustness is crucial for engineering applications, as it ensures stable compensation performance without requiring highly precise and individualized parameter calibration for each sensor, thereby enhancing the method’s practicality and scalability in diverse field conditions.

**Fig 14:**
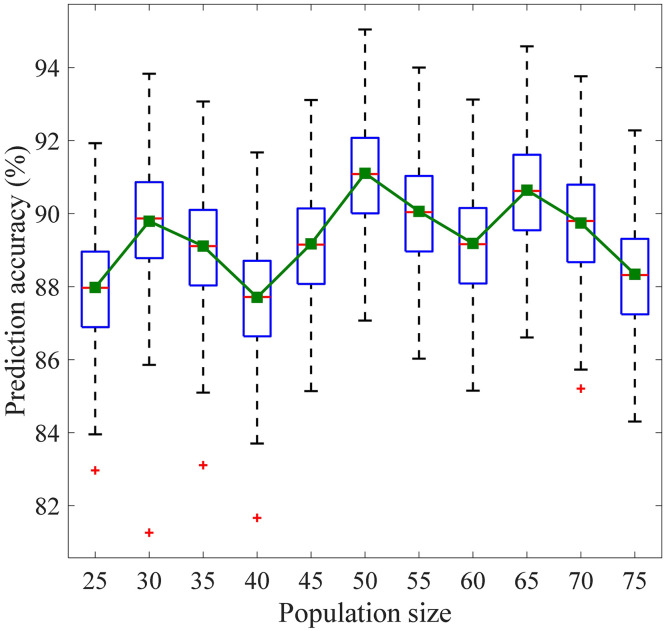
Effect of population size on the prediction accuracy distribution of ratio difference.

Hence, in this section, two important parameters in the Levy-Weighted-QPSO-NN algorithm, population size and maximum iterations, are selected to study their effects on the prediction performance. This can prove that the designed algorithm can still maintain accuracy stability in the case of parameter fluctuation within a certain range, that is, it has the robustness of the algorithm. Fig 14 shows the distribution of prediction accuracy of ratio difference by considering different population size in the proposed Levy-Weighted-QPSO-NN model. Under varying population size parameters, a relatively stable distribution of prediction accuracy was observed. Only when the population size was 25, 30, 35, 40, and 70 did significant outliers in prediction accuracy appear. When the population size changed, the concentration of prediction accuracy and distribution showed a certain degree of decrease in some prediction accuracy distributions. When the population size is 40, the prediction performance is the worst, with an average prediction accuracy of 87.71%.

**Fig 15:**
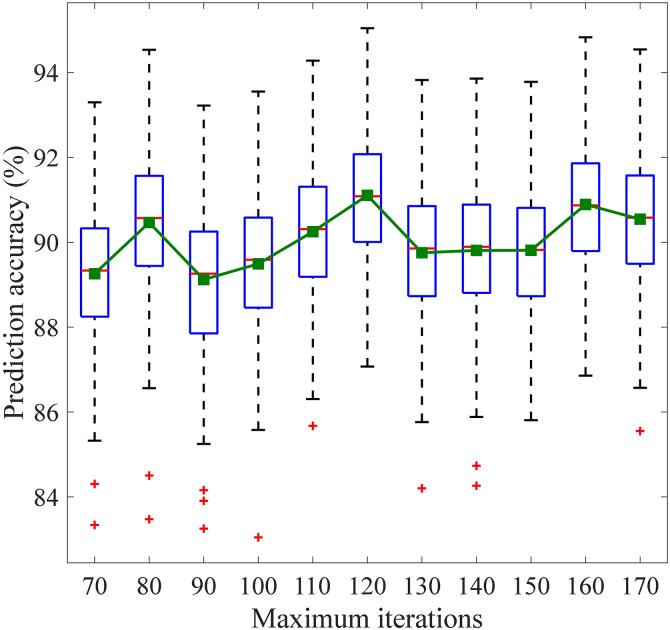
Effect of maximum number of iterations on the prediction accuracy distribution of ratio difference.

Fig 15 shows the distribution of prediction accuracy of ratio difference by considering different maximum iterations in the proposed Levy-Weighted-QPSO-NN model. Compared to the results when the population size changes, the change in prediction accuracy caused by the change in iteration count has a smaller impact. When the number of iterations changes, the prediction accuracy of some parts decreases and the distribution becomes more dispersed, but overall, it still exhibits good prediction performance, indicating acceptable robustness. When the number of iterations is 90, the algorithm’s prediction performance is the worst, with an average prediction accuracy of 89.12% and three outliers in prediction accuracy. Therefore, considering the prediction results under varying parameters of the two algorithms, it can be concluded that the model proposed in this paper possesses robustness.

### 5.4 Temperature-induced error compensation based on prediction results

**Fig 16:**
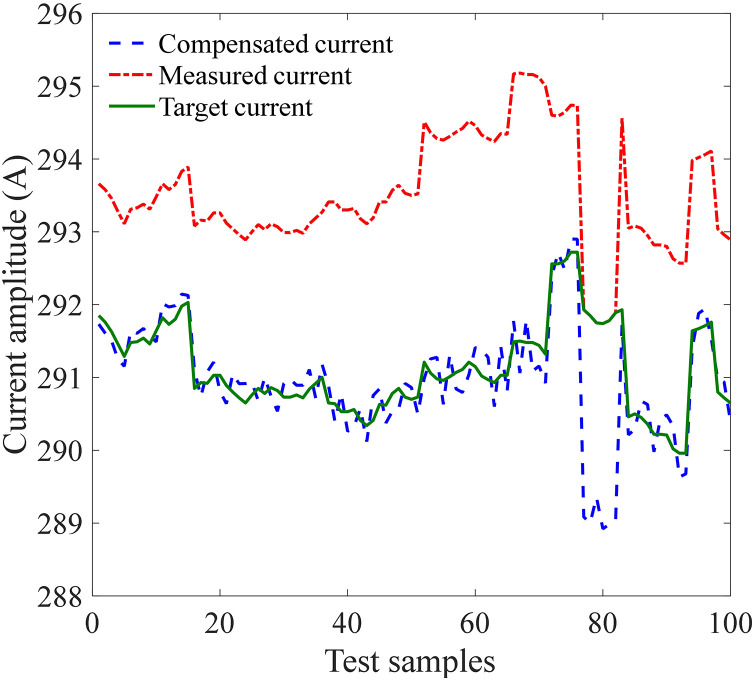
Comparison between the measured current after compensating and initial measured current.

**Fig 17:**
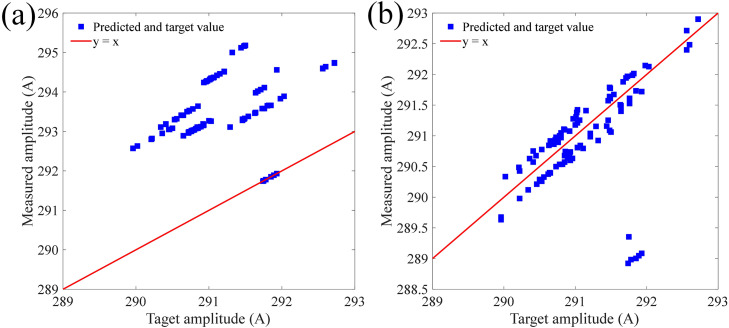
Correlation analysis between target current amplitude and measured current amplitude: (a) before compensation; (b) after compensation.

Fig 16 shows the compensation results based on the predicted ratio difference through Levy-Weighted-QPSO-NN model. It can be seen that, after compensation through Levy-Weighted-QPSO-NN model, the compensated current amplitude is closer to target current amplitude. The MAE before and after compensation is 0.3786 A and 2.3966 A, respectively. The average relative error before and after compensation is 0.82% and 0.13%, respectively. According to the provisions in the reference standards IEC 61869–6/8 [28], GB/T 20840.8−2007 [29], and GB/T 26216.1−2019 [30], the measurement accuracy of optical fiber current sensors is generally required to be Class 0.2S or Class 0.2, which means that the ratio error (ratio difference) limits at the working points of 5%, 20%, 100%, and 120% of the rated current are 0.75%, 0.35%, 0.20%, and 0.20%, respectively. The proposed compensation method can ensure the accuracy requirements of optical fiber current sensors in power grid scenarios under temperature-varying environments. Fig 17 shows the correlation between target current amplitude and measured current amplitude before and after compensation. Combined with the correlation results, it can be concluded that the proposed compensation method based on Levy-Weighted-QPSO-NN model can effectively reduce the measurement error of the fiber optical current sensor caused by temperature changes.

### 5.5 Discussions

In this work, a novel intelligent compensation method for improving the measurement performance of optical fiber current sensor under temperature variations is carried out based on the proposed improved QPSO-NN algorithm. By introducing Levy flight search and weighted average best calculation in traditional QPSO algorithm, the proposed Levy-Weighted-QPSO algorithm is proved to be effective in quicker and more accurate optimization of neural network structural parameters. Therefore, the prediction of measurement ratio difference can be realized based on the measured input variables. Besides, the robustness and stability of the proposed prediction method is also proved to be advantageous compared with other algorithms. Finally, the temperature-induced error compensation shows satisfactory results based on the prediction model method of measurement ratio difference.

While the proposed Levy-Weighted-QPSO-NN model demonstrates high accuracy and robustness in a controlled experimental setting, its transition to real-world engineering applications presents specific challenges, primarily stemming from the ‘black-box’ nature of machine learning models. A key issue for deployment is ensuring the model’s generalization capability across a wider range of sensing rings and environmental conditions beyond those used in training, as performance could degrade due to hardware variances or unforeseen operational scenarios. To address this, future work will focus on developing adaptive online learning mechanisms that allow the model to continuously update its parameters with newly collected field data, thereby maintaining accuracy over the sensor’s lifetime. Furthermore, to mitigate the opacity of the neural network, techniques such as SHAP (SHapley Additive exPlanations) analysis will be employed to interpret the contribution of each input variable, enhancing trust and facilitating fault diagnosis. For practical implementation, the computational efficiency of the algorithm must be optimized, potentially through model simplification or hardware acceleration (e.g., using FPGAs), to meet the real-time processing requirements of power system protection and control. Finally, integrating the compensation for coupled temperature-vibration effects, as preliminarily noted, will be a critical next step to develop a comprehensive error compensation system resilient to the complex multi-stress environment of substations.

This work only considers the influence of temperature on the measurement ratio difference of optical fiber current sensor. It should be noted that, for optical fiber current sensor, the measurement error is not only caused by temperature variations, but also by vibration [31,32]. For example, the opening and closing of high-voltage circuit breaker will lead to obvious impact vibration when the optical fiber current sensor is applied to high-voltage transmission [33]. The influence of temperature and vibration on the measurement error of optical fiber current sensor is often coupled, that is to say, it is difficult to identify the influence of a single factor on the measurement error. In this case, the error compensation method based on intelligent learning can accurately correct the current measurement error when the coupling mechanism is not clear, which exhibits significant engineering significance for improving the actual measurement results of optical fiber current sensor.

Based on the presented work, future research could focus on enhancing the model’s robustness under multi-stress conditions by integrating vibration data as an additional input variable to address the coupled temperature-vibration error mechanism. Furthermore, developing an adaptive online learning framework would be crucial for the model to continuously self-calibrate with long-term field data, ensuring sustained accuracy despite sensor aging or performance drift. For practical deployment, efforts should also be directed towards optimizing the algorithm’s computational efficiency for embedded systems, such as by implementing simplified network structures on FPGA hardware, to meet the real-time processing demands of power system applications.

## 6. Conclusion

In this paper, we proposed an innovate method for compensating measurement error of fiber optical current sensor under temperature variations. The method employs intelligent learning algorithm to accurately predict the measurement ratio difference under the influence of different temperatures, and further utilize the predicted value of the ratio difference to compensate for the measured current amplitude. Main findings and conclusions drawn from this work are summarized as follows.

(1) Faced with the measured error of fiber optical current sensor under temperature variations, we proposed an intelligent compensation method to enhance the measurement effectiveness of the sensor. The neural network-based model is used to construct the nonlinear relationship between easy-to-measure input variables and ratio difference. The predicted difference will be employed to compensate the measured current amplitude.
(2) To enhance the prediction accuracy of the ratio difference of fiber optic current sensors, this paper designs an algorithm framework based on Levy-Weighted-QPSO-NN. Specifically, an innovative Levy-Weighted-QPSO multi-parameter optimization method is designed to obtain the optimal structural parameters of the neural network, in which the Levy flight search and weighted *mbest* calculation method is applied to avoid the problem that traditional optimization algorithms are prone to getting stuck in local optima. The average prediction accuracy of ratio difference of fiber optical current sensor is 91.11%, and the correlation coefficient is 0.9223. After comparative analysis with similar algorithms, the proposed prediction algorithm exhibits significant performance advantages.
(3) The predicted ratio difference based on Levy-Weighted-QPSO-NN algorithm is used to compensate the measured current amplitude under different environmental temperatures. Results show that the proposed method can reduce the measurement error in a temperature-varying environment by 0.69%, achieving an error of 0.13%, which meets the requirements of relevant standards for the measurement accuracy of fiber optic current sensors.

## Supporting information

S1 File Underlying data supporting this work. (ZIP)
